# 2515 Cure Quest: Teaching the complexities of drug discovery and development through an adventure game

**DOI:** 10.1017/cts.2018.202

**Published:** 2018-11-21

**Authors:** Benjamin Chang, Shawn Lawson, Kathleen Ruiz, Mei Si, Jeremy Stewart, Emilia Bagiella, Janice L. Gabrilove, Emma K. Benn

**Affiliations:** RPI, School of Humanities, Arts and Social Sciences

## Abstract

OBJECTIVES/SPECIFIC AIMS: “Cure Quest” is an adventure quest game for mobile tablets that aims to teach the player about the complexities of discovery and development of new medicines. The game instills a sense of wonderment into the learning process, taking the player to a world of magic where a mysterious condition has affected the land and they must follow the steps of the discovery and development process to find a treatment. METHODS/STUDY POPULATION: The game is being developed through a collaboration between faculty and students at ISMMS and the Games and Simulation Arts and Science Program at Rensselaer Polytechnic Institute. The first target audience is 2nd–3rd year medical students, with the future goal of adapting the game to a broader population. RESULTS/ANTICIPATED RESULTS: The game is currently in development, but the project has yielded insight into the design process for serious games in medicine. We found that for a game of this type it is essential not just to have both designers and subject matter experts, but to enable cross-pollination of modes of thinking. Through multiple design iterations and focus groups, we found that a game design approach rooted in narrative and allegorical abstraction would have a better ability to engage the target audience than one focused only on realistic simulation. When complete, we anticipate that the game will improve understanding of the core concepts in drug discovery. DISCUSSION/SIGNIFICANCE OF IMPACT: If successful, the game-based learning approach can help fill key gaps in current formal medical and scientific training, as well as gaps in understanding among the general public. The design process serves as an informative model of evolving collaborative team science.

